# Secondary contacts and genetic admixture shape colonization by an amphiatlantic epibenthic invertebrate

**DOI:** 10.1111/eva.12893

**Published:** 2019-12-03

**Authors:** Jamie Hudson, Kerstin Johannesson, Christopher D. McQuaid, Marc Rius

**Affiliations:** ^1^ School of Ocean and Earth Science National Oceanography Centre Southampton University of Southampton Southampton UK; ^2^ Department of Marine Sciences Tjärnö Marine Laboratory University of Gothenburg Strömstad Sweden; ^3^ Department of Zoology and Entomology Coastal Research Group Rhodes University Grahamstown South Africa; ^4^ Department of Zoology Centre for Ecological Genomics and Wildlife Conservation University of Johannesburg Auckland Park South Africa

**Keywords:** ascidians, gene flow, high‐throughput sequencing, introduced species, introgression, population connectivity

## Abstract

Research on the genetics of invasive species often focuses on patterns of genetic diversity and population structure within the introduced range. However, a growing body of literature is demonstrating the need to study how native genotypes affect both ecological and evolutionary mechanisms within the introduced range. Here, we used genotyping‐by‐sequencing to study both native and introduced ranges of the amphiatlantic marine invertebrate *Ciona intestinalis*. A previous study using microsatellites analysed samples collected along the Swedish west coast and showed the presence of genetically distinct lineages in deep and shallow waters. Using 1,653 single nucleotide polymorphisms (SNPs) from newly collected samples (285 individuals), we first confirmed the presence of this depth‐defined genomic divergence along the Swedish coast. We then used approximate Bayesian computation to infer the historical relationship among sites from the North Sea, the English Channel and the northwest Atlantic and found evidence of ancestral divergence between individuals from deep waters off Sweden and individuals from the English Channel. This divergence was followed by a secondary contact that led to a genetic admixture between the ancestral populations (i.e., deep Sweden and English Channel), which originated the genotypes found in shallow Sweden. We then revealed that the colonization of *C. intestinalis* in the northwest Atlantic was as a result of an admixture between shallow Sweden and the English Channel genotypes across the introduced range. Our results showed the presence of both past and recent genetic admixture events that together may have promoted the successful colonizations of *C. intestinalis*. Our study suggests that secondary contacts potentially reshape the evolutionary trajectories of invasive species through the promotion of intraspecific hybridization and by altering both colonization patterns and their ecological effects in the introduced range.

## 1 INTRODUCTION

It is well established that attributes of nonindigenous species (NIS) such as genetic diversity (Dupont, Jollivet, & Viard, 2003), founder group size (Lockwood, Cassey, & Blackburn, 2005), inbreeding depression (Roman & Darling, 2007) and genetic admixture (Verhoeven, Macel, Wolfe, & Biere, 2011) influence their colonization success. These attributes are not mutually exclusive and often combine to allow or deter species introductions (Rius, Turon, Bernardi, Volckaert, & Viard, 2015). In addition, genetic data are critical for (a) reconstructing invasion routes, (b) identifying the source population(s) and (c) understanding how anthropogenic factors affect colonization success (Cristescu, 2015; Estoup & Guillemaud, 2010). Despite a great deal of recent research on invasion genetics (Bock et al., 2015; Bourne, Hudson, Holman, & Rius, 2018; Rius et al., 2015), there remains a dearth of studies investigating how genetic patterns in the native range influence the introduced range.

Biological invasions act as unique experiments in evolution (Yoshida, Goka, Ishihama, Ishihara, & Kudo, 2007), allowing observations of how NIS spread and adapt to novel environments on a human timescale. The genetic study of NIS furthers our understanding on how contemporary gene flow and local adaptation contribute to colonization success (Verhoeven et al., 2011). In addition, studies of NIS have shown that genetic admixture of divergent lineages can affect fitness of colonizing populations through transgressive segregation (Johansen‐Morris & Latta, 2006; Wagner, Ochocki, Crawford, Compagnoni, & Miller, 2017), by masking deleterious mutations (Keller & Waller, 2002), and/or by increasing standing genetic variation on which selection can act (Rius & Darling, 2014). Genetic admixture can also disrupt locally adapted gene pools, which may negatively affect colonization success (Gilk et al., 2004). Therefore, understanding how ecological and evolutionary mechanisms influence colonization success is key for unravelling how genetic patterns found in native and introduced ranges relate. Research progress on the evolutionary effects of NIS has largely been dominated by studies conducted in terrestrial ecosystems (Abbott, 1992; Rius & Darling, 2014), with considerably less effort being devoted to study aquatic organisms.

Ascidians (Chordata, Tunicata, Ascidiacea) are marine sessile invertebrates that are notoriously invasive (Lambert & Lambert, 1998) and frequently foul aquaculture facilities (Fitridge, Dempster, Guenther, & de Nys, 2012; Rius, Heasman, & McQuaid, 2011) and marine infrastructures (Johnston, Dafforn, Clark, Rius, & Floerl, 2017). The early life‐history stages of ascidians are ephemeral and represent the only dispersive stages of their life cycle (Millar, 1971), offering only highly restricted natural dispersal. Thus, long‐distance dispersal of ascidians is attributed to artificial transport (Hudson, Viard, Roby, & Rius, 2016) or rare rafting events (Carlton et al., 2017). As such, they are relevant and unique models for studying colonization success in marine ecosystems (Zhan, Briski, Bock, Ghabooli, & MacIsaac, 2015). *Ciona intestinalis* is a solitary ascidian with a disjunct amphiatlantic (i.e., inhabiting both sides of the Atlantic) distribution throughout the North Atlantic Ocean (Bouchemousse, Bishop, & Viard, 2016a). It is generally accepted that the northeast Atlantic coastline is its native range (Bellas, Beiras, & Vázquez, 2003; Bouchemousse, Bishop, et al., 2016a; Gulliksen & Skjæveland, 1973; Nydam et al., 2017), while the introduced range includes the northwest Atlantic coastline (Bouchemousse, Bishop, et al., 2016a; Nydam & Harrison, 2007). As with all solitary ascidians, *C. intestinalis* is hermaphroditic and reproduces through broadcast spawning, with external fertilization. The short‐lived pelagic larval stage normally lasts <24 hr, though this stage can be extended to five days (Petersen & Svane, 1995). Larvae of *C. intestinalis* are often retained close to the adults and the production of adhesive mucus strings together with the eggs (Svane & Havenhand, 1993) may result in lower dispersal potential. Consequently, transcontinental dispersal of *C. intestinalis* is attributed to anthropogenic transport or rafting events of individuals only. *Ciona intestinalis* shows a high affinity for marine infrastructures (e.g., pontoons and ropes in harbours and marinas), which are known to concentrate NIS (Aldred & Clare, 2014). This propensity to foul can lead to negative economic and ecological impacts when this species is found in aquaculture facilities (Fitridge et al., 2012; Lutz‐Collins, Ramsay, Quijón, & Davidson, 2009; Rius et al., 2011). Consequently, most research studying the extensive distribution of *C. intestinalis* has been performed considering individuals found on artificial structures (e.g., Bouchemousse, Bishop, et al., 2016a; Bouchemousse, Liautard‐Haag, Bierne, & Viard, 2016c; Hudson et al., 2016; Zhan, Macisaac, & Cristescu, 2010). This has led to a good understanding of the distribution of *C. intestinalis* on artificial structures, but there is still limited knowledge of the relative importance of natural and artificial habitats for the spread and establishment of this species in new areas.

The west coast of Sweden is a coastline where *C. intestinalis* is present on natural substrata from the surface to depths of more than 100m (Dybern, 1965, 1967; Svane & Havenhand, 1993). There, the opening of the brackish waters of the Baltic Sea to the Atlantic means that individuals inhabiting shallow water experience a wide range of salinities (10–30 PSU) and variable temperatures (~0–20°C, Dybern, 1965; Renborg, Johannesson, & Havenhand, 2014), whereas individuals at depth live in both more constant temperatures and stable, high salinities (~34 PSU). The difference in density between surface and deeper waters leads to a strong pycnocline separating the less saline surface water of the Baltic Sea from the high salinity bottom water (often more than ~10‐15 m in depth) from the Atlantic (Johannesson et al., 2018). There are observable differences in the biology and life history of individual *C. intestinalis* found in different depths. For example, individuals inhabiting shallow waters (<15 m) have two generations a year (each spawning period lasting a couple of months) during boreal spring and late summer, whereas deeper individuals (>15 m) have one generation per year, with spawning lasting approximately one month during boreal summer (Dybern, 1965). Additionally, there appears to be slight morphological variation across depths, with shallow individuals being smaller and more heavily pigmented than deeper individuals (Dybern, 1965; Svane, 1983). This may be due to genetically driven phenotypic variation. A recent study using microsatellites showed that the deep and shallow water populations of *C. intestinalis* along the Swedish west coast are genetically differentiated (Johannesson et al., 2018). Strong pycnoclines can act as distinct barriers to vertical movement of larvae within the water column (e.g., Gallager, Davis, Epstein, Solow, & Beardsley, 1996), and the existence of genetically distinct populations has tentatively been ascribed to the pycnocline present at ~10‐15 m acting as a barrier to reproductive exchange. In addition, local adaptation may contribute to the genetic differences between shallow and deep populations as they are exposed to different conditions, including salinity, temperature, food availability and light. Thus, two distinct populations of *C. intestinalis* separated by an abiotic barrier have evolved along the Swedish west coast.

Here, we used *C. intestinalis* as a model organism to investigate how understanding genetic variability in the native range can help elucidate mechanisms shaping both colonization success and introduction pathways in new ranges. The objectives of the study were to (a) identify fine‐ and broad‐scale population genomic patterns of *C. intestinalis*, (b) reveal evolutionary relationships among individuals collected along coastlines across the range of the species, (c) determine the presence or not of genetic admixture and (d) if admixture was present, infer if it could be associated with successful colonization of novel habitats. We hypothesized that the colonization success of *C. intestinalis* across its introduced range has been affected by the historic divergence of ancestral genotypes, the levels of genetic admixture between divergent lineages, and the intensity of gene flow between native and introduced ranges.

## 2 MATERIALS AND METHODS

### 2.1 Field sampling

**Figure 1:**
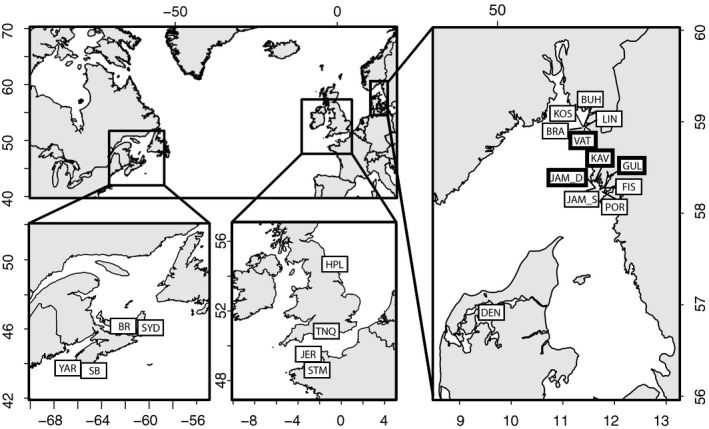
North Atlantic coastlines where the samples of *Ciona intestinalis* were collected. Sampling site names are abbreviated as in Table 1, with bold quadrats around site codes representing deep sampling sites. The putative native range in the literature includes Scandinavia, the British Isles and the English Channel, whereas the introduced range includes the northwest Atlantic

Tissue samples of 285 *C. intestinalis* were collected from 20 sites within the putative native and introduced range of the species (Table 1 and Figure 1). Samples from Sweden were collected from shallow natural, deep natural, and shallow artificial substrata (see details in Table 1), whereas sites outside Sweden were all from shallow artificial substrata. Individuals from natural substrata were sampled by either snorkelling, SCUBA diving or a remotely operated underwater vehicle. Artificial substrata were sampled in marinas by pulling up hanging ropes, submerged buoys and checking the undersides of pontoons. We attempted to leave a distance of at least one metre between each sampled individual to limit the chance of collecting closely related individuals. Once collected, tissue was immediately preserved in 95% ethanol which was periodically replaced until tissue pigment no longer leached into the ethanol. Finally, tissue samples were stored at −20°C until DNA extraction.

**Table 1:** Sampling information for *Ciona intestinalis*

Country	Site name	Code	Latitude (N)	Longitude (E or W)	Depth (category)	Substratum	No. of individuals sequenced	*F*_IS_	H_E_
Sweden	Vattenholmen	VAT	58.87°	11.09°	60 m (Deep)	Natural	16	0.065	0.218
	Gåseklåvan	GUL	58.31°	11.54°	20–25 m (Deep)	Natural	15	*0.121*	0.267
	Jämningarna	JAM_D	58.26°	11.39°	17–20 m (Deep)	Natural	8	0.086	0.286
	Kåvra	KAV	58.33°	11.36°	18–22 m (Deep)	Natural	16	*0.113*	0.230
	Burholmen	BUH	58.89°	11.13°	5 m (Shallow)	Natural	16	*0.139*	0.202
	South Koster	KOS	58.88°	11.05°	3–4 m (Shallow)	Natural	15	0.078	0.232
	Brattskär	BRA	58.86°	11.07°	1–4 m (Shallow)	Artificial	15	*0.078*	0.238
	Lindholmen	LIN	58.88°	11.15°	0–1 m (Shallow)	Artificial	14	0.088	0.242
	Porsholmen	POR	58.23°	11.40°	2–4 m (Shallow)	Natural	15	0.056	0.233
	Jämningarna	JAM_S	58.26°	11.39°	5–7 m (Shallow)	Natural	16	0.028	0.206
	Fiskebäckskil	FIS	58.24°	11.46°	0.5–2 m (Shallow)	Artificial	15	0.073	0.241
Denmark	Limfjord	DEN	56.78º	9.18º	0.5–2 m (Shallow)	Artificial	20	*0.095*	0.224
England	Hartlepool	HPL	54.69º	−1.20º	0.5–2 m (Shallow)	Artificial	18	*0.089*	0.227
	Town Quay	TNQ	50.89º	−1.41º	0.5–2 m (Shallow)	Artificial	12	*0.121*	0.231
Jersey	St. Helier	JER	49.18º	−2.12º	0.5–2 m (Shallow)	Artificial	15	*0.082*	0.217
France	St. Malo	STM	48.64º	−2.03º	0.5–2 m (Shallow)	Artificial	14	*0.117*	0.232
Canada	Yarmouth	YAM	43.83º	−66.13º	0.5–2 m (Shallow)	Artificial	12	0.069^a^	0.229^a^
	Shelburne	SB	43.76º	−65.32º	0.5–2 m (Shallow)	Artificial	2		
	Brudenell River	BR	46.20º	−62.59º	0.5–2 m (Shallow)	Artificial	9	0.017^b^	0.257^b^
	Sydney	*SD*	46.14º	−60.19º	0.5–2 m (Shallow)	Artificial	2		
							265		

Note

The table includes geographical region, site name abbreviation (Code), coordinates of sampling sites, depth (shallow [<15 m] or deep [>30 m]), substratum collected from and the number of individuals used in genomic analyses. Additionally included are inbreeding coefficient *F*_IS_ values (values in italics are statistically significant [*p* < .05]) and population mean expected heterozygosity (H_E_).

^a^ Refers to merged samples known as CAN_1 (Yarmouth and Shelburne).

^b^ Refers to merged samples known as CAN_2 (Brudenell River and Sydney).

### 2.2 DNA extraction and genotyping

DNA was extracted from preserved tissue using the Qiagen DNeasy^®^ Tissue Kit (Qiagen) according to the manufacturer's protocol. Gel electrophoresis and the QuantiFluor^®^ dsDNA System (Promega) were used to assess quality and quantity of extracted DNA, respectively. DNA was shipped to the University of Wisconsin Biotechnology Center where it was genotyped using the genotyping‐by‐sequencing methodology (GBS; Elshire et al., 2011). Briefly, GBS reduces the complexity of the sample genome by digesting the DNA using methylation‐sensitive restriction enzymes and sequencing the ends of the digested fragments using barcoded adapter regions.

### 2.3 Analysis of genotyping‐by‐sequencing data

The GBS assembly was performed using ipyrad v. 0.7.28 (Eaton, 2014), a toolbox for assembly and analysis of restriction site‐associated DNA sequencing (RAD‐seq) type genomic data sets. We followed the seven sequential assembly steps of ipyrad using parameters based on those recommended for single‐end GBS data in the ipyrad documentation (http://ipyrad.readthedocs.io/). As the *C. intestinalis* genome is not yet available, we used the de novo assembly method, which requires no prior genomic resources and used ipyrad to trim Illumina adapter reads. As we were working with only one species, we set the level of sequence similarity for clustering to be 90% (I. Overcast, pers. comm.). Following the iterative filtering framework outlined by O'Leary, Puritz, Willis, Hollenbeck, and Portnoy (2018), we used vcftools v.0.1.13 (Danecek et al., 2011) to first filter for loci with a minimum single nucleotide polymorphism (SNP) call quality of 20, a minimum genotype depth of less than five, and a mean minimum depth (across individuals) of <15. Additionally, we chose to remove loci with a minor allele count of less than three, rather than the commonly used minor allele frequency threshold of 5%, because the latter will remove true rare alleles that are important in elucidating fine‐scale structure and accurately drawing inference of past demographic events (O'Connor et al., 2015). We then iteratively increased our stringency for allowing missing data (on both loci and individuals separately), so that our final dataset contained loci with at least 50% call rate (i.e., a locus must be present in at least 50% of individuals), and up to 50% allowed missing data per individual. To remove the confounding effects of linkage disequilibrium, we used vcftools to thin markers so that only one SNP per locus was retained in our dataset.

We used BayeScan v.2.0 (Foll & Gaggiotti, 2008) to ensure our dataset contained only putatively neutral loci. BayeScan uses differences in allele frequency between populations to identify candidate loci under natural selection (Foll & Gaggiotti, 2008) and was run using a thinning interval size of 20, with 25 pilot runs of length 10,000 and a burn‐in length of 50,000. Prior odds for the neutral model were set to 100 rather than the default 10, to reduce the number of false positives in large datasets (>1,000 SNPs). While commonly used, BayeScan has often been shown to report false positives especially in species undergoing range expansions, while also assuming equal population exchange and evolutionary independence among all populations (Bierne, Roze, & Welch, 2013; Whitlock & Lotterhos, 2015). We therefore also assessed for candidate loci using two newer methods, *OutFlank* v.0.2 (Whitlock & Lotterhos, 2015) and pcadapt v.4.1.0 (Luu, Bazin, & Blum, 2017). Similarly to BayeScan, *OutFlank* groups individuals into predefined populations, before inferring candidate loci based on a trimmed distribution of *F*_ST_ values for loci deemed to be neutral. As reported in similar studies (see results and Guzinski, Ballenghien, Daguin‐Thiebaut, Leveque, & Viard, 2018), *OutFlank* did not recover any *F*_ST_ outlier loci, so we continued our analyses with other software. Regarding the pcadapt, it ascertains population structure using principal component analysis (PCA) to find candidate loci excessively related to population structure. We classified loci that were recovered by both BayeScan and pcadapt as putatively under natural selection and removed them for the following analyses. Finally, we created a more conservative dataset that excluded all loci recovered by BayeScan and pcadapt and ran the whole set of analyses again (see Appendix A within Supporting Information).

### 2.4 Population structure

We used the software ADMIXTURE v.1.3 (Alexander, Novembre, & Lange, 2009) to estimate the likelihood that an individual comes from one of a certain number of putative sample populations (K). Like STRUCTURE v.2.3.4 (Pritchard, Stephens, & Donnelly, 2000), ADMIXTURE uses a maximum‐likelihood estimation from multilocus SNP genotype datasets, but calculates estimates using a faster numerical optimization algorithm. We performed a discriminant analysis of principal components (DAPC) to visualize between‐population genomic variation (Jombart, Devillard, & Balloux, 2010). DAPC transforms the data using PCA before using PCA factors as variables for a discriminant analysis (DA), ultimately maximizing the differences among groups while minimizing variation within groups (Jombart et al., 2010). We used the package *adegenet* v.2.1.1 (Jombart, 2008) for R (R Development Core Team, 2017) to perform the DAPC. We ran the DAPC with and without a priori knowledge of individual populations. We examined pairwise population genetic differentiation using *F*_ST_ values and their *p* values by running 10,000 permutations with Arlequin v.3.5 (Excoffier & Lischer, 2010). We also used Arlequin to measure the inbreeding coefficient *F*_IS_ and expected heterozygosity (H_E_) per population. Finally, we ran an analysis of molecular variance (AMOVA) test using site clusters as inferred by ADMIXTURE and DAPC plots and also using only shallow Sweden sites to test whether there was an effect of substratum (natural vs. artificial). AMOVAs were performed in Arlequin v.3.5. (Excoffier & Lischer, 2010).

### 2.5 Reconstructing invasion routes

To obtain relevant and detailed information and infer the historical relationship among genotypes of *C. intestinalis* throughout its range, we analysed sets of evolutionary scenarios with the approximate Bayesian computation (ABC) method using DIYABC v.2.0.1 (Cornuet et al., 2014). We grouped sites based on their geographical location and the results of above population structure analyses (shallow Sweden sites plus the Denmark site, deep Sweden sites, England, Jersey and France sites, and Canada sites). The high shipping traffic between the native and introduced ranges (Kaluza, Kölzsch, Gastner, & Blasius, 2010), coupled with the similarity in genetic diversity across our sampled sites (see Results), meant we did not consider the presence of genetic bottlenecks while designing the evolutionary scenarios. Our first two sets of scenarios aimed to infer the evolutionary history within the northeast Atlantic (see details of scenario sets 1 and 2 in Figures S3a,b, respectively). Following the results of this initial analysis, we then added in data from Canada sites to infer the colonization history along the introduced range (scenario set 3, Figure S3c). As specific population sizes, divergence times and potential admixture rates were unknown, we used a uniform distribution with a large interval (population sizes and divergence times: 10–10^7^; admixture rates: 0.001–0.999; Table S5) when setting priors for each parameter (White, Reyes‐Betancort, Chapman, & Carine, 2018). We used the mean genic diversity, mean distribution of *F*_ST_ distances, mean distribution of Nei distances, and whenever an admixture event was included in the scenario, mean admixture estimates for summary statistics. For all scenarios, we used the default 10^6^ simulated data per scenario to build reference tables. Upon creation of the reference table, we pre‐evaluated scenarios and prior distributions by performing a PCA in the space of the summary statistics on 1,000 simulated datasets for each scenario and adding the observed dataset to each plane (Cornuet et al., 2014). We used a logistic regression on the 1% simulated datasets that were closest to the observed dataset (using Euclidean distances between simulated and observed datasets) to calculate the posterior probability of each scenario. This approach produces 95% confidence intervals for each scenario's posterior probability, with the most likely scenario defined as the highest estimate without overlapping confidence intervals (Cornuet et al., 2008). For the most probable scenario of scenario set 3 (Figure S3c), we calculated type I (the probability with which this scenario is rejected although it is the true scenario) and type II (the probability of choosing this scenario when simulating data according to other scenarios) error rates. Finally, we assessed the goodness of fit for the final chosen scenario by implementing the model checking feature of DIYABC. We simulated 1,000 datasets using posterior distribution values and compared these with the observed dataset by considering different summary statistics than were used during the generation of the reference table, and visualized this using a PCA (Cornuet et al., 2014).

## 3 RESULTS

### 3.1 Loci assembly and detection of outlier loci

GBS generated a total of 530,157,826 raw reads, with an average of 2,000,596 reads per sample. After filtering and clustering using ipyrad and vcftools, we retained a total of 1,667 putatively unlinked SNPs in the sequence assembly. Twenty individuals were removed from the dataset due to missing data (i.e., >50% missing data), which was likely caused by poor DNA quality or secondary contaminants within the samples (Federman, Donoghue, Daly, & Eaton, 2018). This led to a final dataset of 265 individuals from 20 separate sampling sites. However, the Canadian sites were merged as CAN_1 (sites Yarmouth and Shelburne) and CAN_2 (Brudenell River and Sydney) due to the limited number of individuals obtained from Shelburne and Sydney. Therefore, the total final number of sites was 18. BayeScan and pcadapt recovered a total of 30 and 61 *F*_ST_ outlier loci, respectively, of which 14 were found by both software, whereas *OutFlank* recovered no putative loci under selection. We subsequently removed the 14 loci found in both BayeScan and pcadapt from our analyses, leaving a dataset of 1,653 SNPs. We also performed all analyses on a new dataset that excluded all *F*_ST_ outlier loci recovered, irrespective of program used (77 loci in total, see Appendix A within the Supporting Information for details).

### 3.2 Heterozygosity and population structure

Values of *F*_IS_ ranged from 0.017 to 0.139 (Table 1). Nine sites showed no signs of deviation from Hardy–Weinberg equilibrium, but nine of the sites exhibited significant positive *F*_IS_ values indicating a deficiency of heterozygotes in these sites (Table 1). Expected heterozygosity ranged from 0.202 to 0.286 (Table 1), with no noticeable differences in genetic diversity between geographical regions (Table 1).

**Figure 2:**
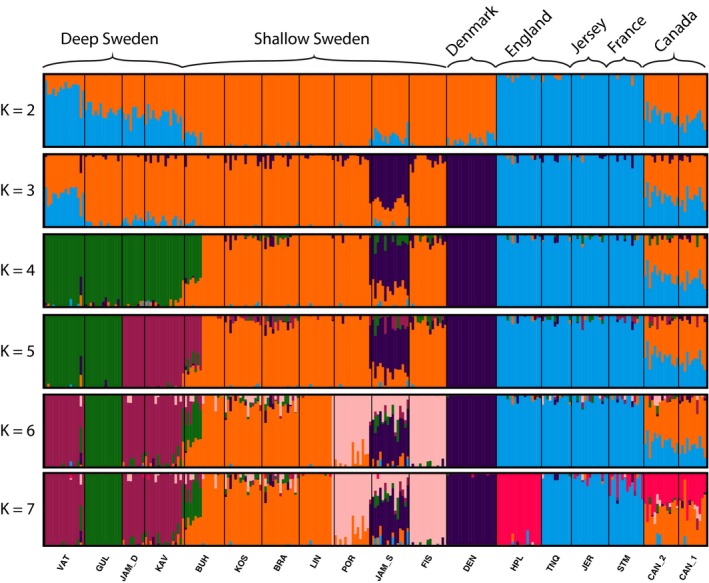
ADMIXTURE plots representing all sampled populations of *Ciona intestinalis*. The main regions are highlighted above. The different colours represent putative genetic clusters with *K* ranging from 2 to 7, with *K* = 4 being found to be the most optimal value (Figure S1). Sampling site names are abbreviated as in Table 1

The combination of ADMIXTURE, DAPC and pairwise site comparisons of *F*_ST_ allowed us to identify fine‐ and broad‐scale population genomic patterns. Cross‐validation by ADMIXTURE inferred the most likely number of sampled populations was *K* = 4 (Figure S1) and broadly indicated the structuring of deep Sweden sites (green in Figure 2), shallow Sweden sites (orange) and those found in England, Jersey and France (blue). Individuals from Canada appeared to have a genetic background similar to both individuals found in England, France, Jersey and individuals from shallow Sweden. The Denmark samples clustered with a shallow Sweden site (shallow Jämningarna, purple cluster), and eight individuals from the shallow Sweden site Burholmen were grouped with samples from deep Sweden.

**Figure 3:**
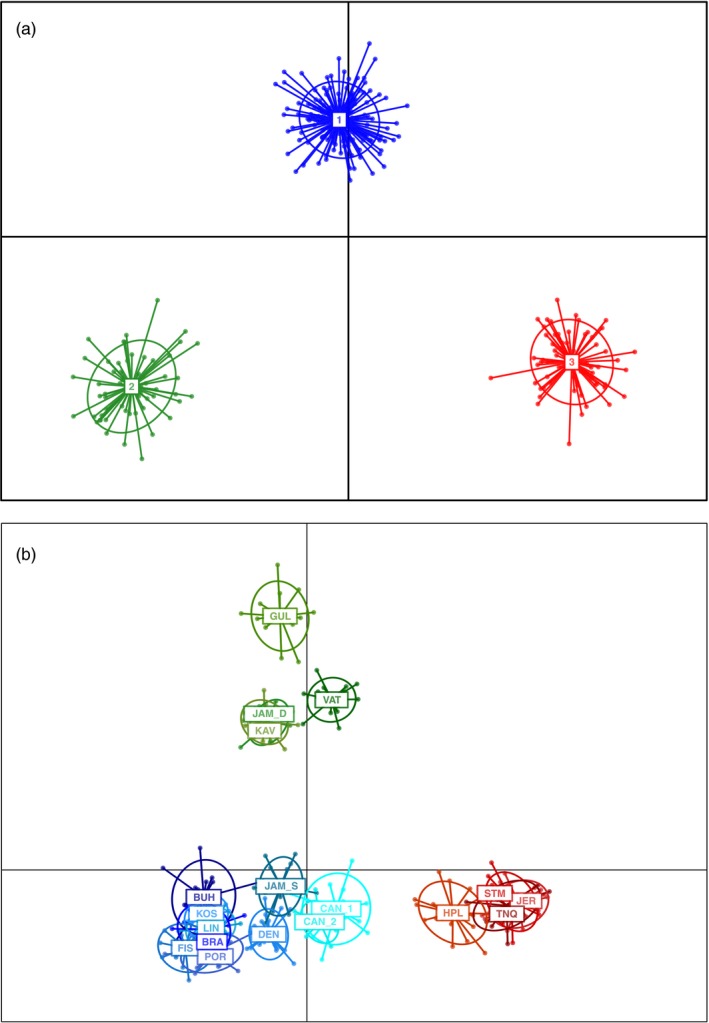
(a) Discriminant analysis of principal components using unlinked loci with no a priori population information. The first axis explains 58.2% of the variation, and the second axis explains 41.8%. (b) Discriminant analysis of principal components using unlinked loci with a priori population information. The first axis explains 23.1% of total variation, and the second axis explains 18.1%. Sampling site names are abbreviated as in Table 1. Sites in (a) are assigned to clusters as follows; Cluster 1: FIS, KOS, BRA, LIN, POR, BUH, JAM_S, DEN, CAN_1, CAN_2, and eight individuals from BUH; Cluster 2: JER, TNQ, HPL, STM; Cluster 3: VAT, JAM_D, GUL, KAV, and eight individuals from BUH

The ADMIXTURE patterns were supported by the DAPC analysis with and without prior sample assignment (Figure 3a,b), which recovered three genetic clusters, one of which (cluster 3) included the shallow Sweden sites (except the eight individuals from BUH) and sites from Denmark and Canada, and the other two clusters including deep Sweden sites and sites from England, Jersey and France, respectively.

**Figure 4:**
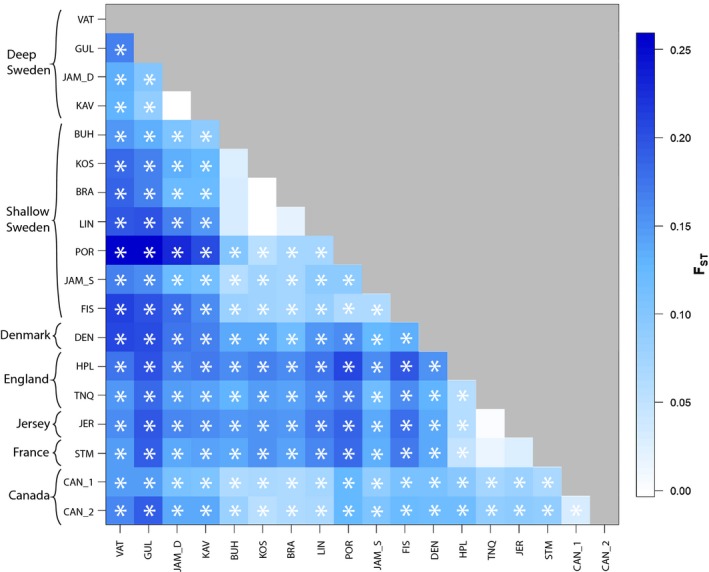
Matrix of *F*_ST_ values. Asterisks represent significant values, after Bonferroni correction. Sampling site names are abbreviated as in Table 1

Pairwise comparisons of *F*_ST_ suggested very strong genetic structuring among most sites (Figure 4), with 143 out of 153 comparisons (93%) being significant, including clear structuring between shallow and deep Sweden sites (Figure 4, Table S1). Notably, there was significant genetic differentiation among the deep Sweden sites with the exception of two deep sites (Kåvra vs. deep Jämningarna) that are very close to one another. Pairwise site comparisons among shallow Sweden sites found 15 of 21 comparisons (71%) were significant, while comparison of the two Canadian sites provided a low, but significant, *F*_ST_ value (Figure 4, Table S1).

The AMOVA test using site clusters as inferred by ADMIXTURE and DAPC plots showed that genetic differentiation was significant among groups, among sites within groups and within sites (Tables S2,S3). The AMOVA test performed using only shallow Sweden sites to test whether there was an effect of substratum (natural vs. artificial) found no significant genetic differentiation between these two groups (Table S4).

### 3.3 Reconstructing invasion routes

**Figure 5:**
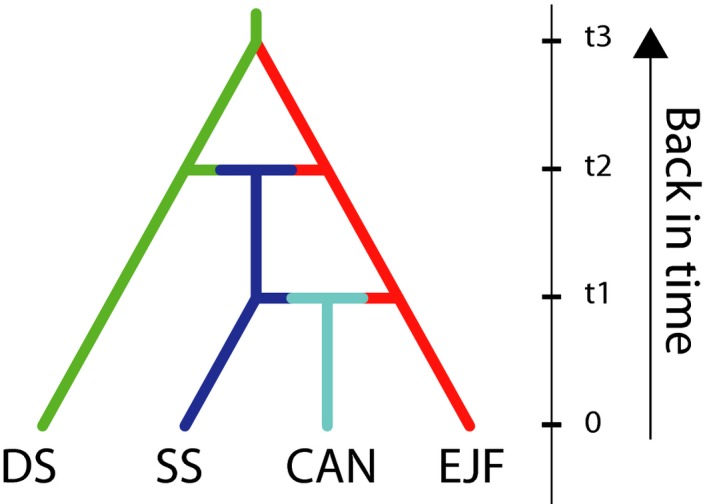
The most likely evolutionary scenario involving the sample sites as calculated using DIYABC. The y‐axis represents time (not to scale). Included are shallow Sweden sites (SS), Canada sites (CAN), the Denmark site (DEN), England, Jersey, and France sites (EJF), and deep Sweden sites (DS). The full set of scenarios can be found in Figure S3

For all of our ABC analyses, our check of priors showed a good match between simulate datasets and the observed data (Figure S2). We firstly found that within our northeast Atlantic sampling sites, the ancestral population diverged and formed the deep Sweden and England, Jersey, France groups (Figure S3a), with the logistic estimate of posterior probability for this scenario being *p* = .996 (CI = 0.994, 0.997; Table S6). For our next set of scenarios, which assessed the origin of the shallow Sweden group (Figure S3b), we found the scenario with the highest support being an admixture event between deep Sweden and England, Jersey, and France groups (*p* = .998, CI = 0.998–0.999; Table S6) following secondary overlap of the two lineages. Our final set of scenarios, which assessed the scenario that best explains the colonization of the introduced range (Figure S3c), found that the most likely was a recent admixture between shallow Sweden sites and English, Jersey and France sites (*p* = .841. CI = 0.832, 0.851; Table S6; Figure 5). The type I error rate was 0.15, showing that 85% of our datasets simulated with the highest supported scenario (Figure 5) were correctly identified as being produced by the same scenario. Moreover, type II error rate was on average 0.04. Our model checking procedure for the most likely scenario found that for the 57 summary statistics used for model checking, 23 different significantly from the simulated distribution (Table S7, Figure S4), suggesting that even though this is the most strongly supported scenario, there is some discordance between the scenario posterior combinations and the observed dataset.

## 4 DISCUSSION

Our results showed high levels of genomic differentiation between the main regions of the northeast Atlantic (i.e., English Channel and North Sea) and identified the presence of historical genetic admixture among individuals from these regions. This seems to have resulted in genotypically and phenotypically distinctive individuals that are currently found in shallow sites in Sweden. In addition, we revealed genomic patterns suggesting secondary contacts and postulate that this may have promoted intraspecific hybridization. Our result supported the presence of genetic admixture during the spread to and colonization of the northwest Atlantic. More specifically, we found evidence of genetic admixture between genotypes from the English Chanel and genotypes from the shallow North Sea. While we found here no direct evidence that intraspecific hybridization influences colonization, our results indicate that this may be a possible mechanism promoting successful colonization of sites with new environmental conditions, such as trans‐oceanic introductions. This builds on a growing number of studies showing that the mixing of divergent genotypes as a result of human mediated transport of species has the potential to fundamentally alter colonization patterns and to unprecedentedly alter ecological and evolutionary patterns (Bouchemousse, Liautard‐Haag, et al., 2016c; Mooney & Cleland, 2001; Pineda, López‐Legentil, & Turon, 2011).

The presence of high genetic subdivision among genotypes found in deep sites off Sweden and in England, Jersey and France suggests that individuals found in these sites represent native populations (Figure 3). This is supported by the ABC analyses, which indicated an initial divergence between these two groups (Figure 5). This accords with the expectation that native ranges will show a highly defined population structure, often involving two main groups of ancestral genotypes (Boubou et al., 2012; Reusch, Bolte, Sparwel, Moss, & Javidpour, 2010; Rius, Clusella‐Trullas, et al., 2014a). Divergence of these *C. intestinalis* populations may reflect adaptation to differing local conditions and/or earlier periods of allopatric isolation leading to the generation of genetic divergence through selection or genetic drift. Previous research has shown that high plasticity in *C. intestinalis* allows acclimatization of deep water individuals to shallow water salinities (Renborg et al., 2014), which suggests that local adaptation and primary divergence are less likely. Rather, it seems more likely that during the last glacial maximum deep sites off Sweden and sites in England, Jersey and France were isolated as separate glacial refugia, leading to the divergence that we see today. ABC analyses suggest that secondary contacts leading to genetic admixture between the England, Jersey, France and the deep Sweden genotypes formed the genotypes found in shallow Sweden and Denmark. Thus, either historic artificial transport or postglacial expansion may have promoted such secondary contacts, as reported for other marine invertebrates (Pérez, Nirchio, Alfonsi, & Muñoz, 2006). A similar situation has been identified with the ascidian *Pyura chilensis* in the southwest Pacific Ocean, where historical divergence of *P. chilensis* populations occurred due to isolation associated with glacial periods. This was followed by secondary contacts and genetic admixture between these previously isolated populations (Haye & Muñoz‐Herrera, 2013). Our results suggest that genetic admixture may have had fitness effects that enabled *C. intestinalis* to expand to previously uninhabitable substrata and conditions within its native range. The ability of individuals from shallow Sweden populations to survive relatively high temporal variability in environmental conditions such as temperature and salinity compared to individuals found in England, Jersey, France and deep Sweden may be explained by the fitness benefits of genetic admixture (Wagner et al., 2017), allowing survival in the face of strong selective pressures (Verhoeven et al., 2011).

In contrast to the Sweden populations, individuals from England, Jersey and France formed a relatively homogeneous genetic cluster in both ADMIXTURE and DAPC analyses (Figures 2, 3, 4). Earlier work showed that samples from these locations were subdivided into two genetic groups (Hudson et al., 2016), but this differentiation was weaker than what we found between samples from deep Sweden and those from England, Jersey and France. The native range of *C. intestinalis* has been previously described as the northeast Atlantic (e.g., Bouchemousse, Bishop, et al., 2016a; Hudson et al., 2016), and here, we show that this range comprises most of the genomic differentiation among populations, with more complex demographic histories among populations along the northeast coast of the Atlantic than the northwest coast. In line with previous studies that identified admixture within native ranges (Gillis, Walters, Fernandes, & Hoffman, 2009; Rius, Turon, Ordóñez, & Pascual, 2012), our findings suggest that historic artificial transport may have facilitated the admixture of the genotypes from deep Sweden and the English Channel.

It is well established that the recent spread of *C. intestinalis* has been promoted by the proliferation of man‐made structures along coastlines that act both as stationary substrata and as vectors (Clarke Murray et al., 2014). Throughout the study area, this species is widespread in harbours and marinas in much of its current distribution (Bouchemousse, Bishop, et al., 2016a; Hudson et al., 2016), but rare on natural substrata except along the Swedish coast (Johannesson et al., 2018). This raises the issue of the origin and evolutionary background of the many populations living on artificial substrata that may represent either extensions of large natural populations or completely new introduced populations. In the sampled shallow Sweden sites, comparison of genetic differentiation between natural and man‐made sites showed no significant differences (Table S4), with individuals sampled on artificial substrata being generally more closely related to nearby shallow natural sites than to individuals from other artificial sites along the Swedish coast (Figure 4; Table S1). This suggests that the nature of natural and artificial substrata does not, in itself, create a barrier to local gene flow. In shallow waters off Sweden, the sampled natural substratum included seagrass beds where *C. intestinalis* lives at modest densities attached to blades of *Zostera marina*. Below the pycnocline, at depths of 20 m or more, dense populations occur on the natural vertical rock walls (see also Svane & Havenhand, 1993), whereas in the English Channel and south‐western North Sea area, very few individuals have been documented on natural substrata. To our knowledge, there are no reports of *C. intestinalis* inhabiting natural substrata in the English Channel. However, small numbers have been recovered during dredging estuaries in the English Channel (authors pers. obs.) and the south‐western North Sea (Rees, Waldock, Matthiessen, & Pendle, 2001). Such low densities may be due to the effects of predation on different life‐history stages as seen for closely related species in other parts of the world (Dumont, Gaymer, & Thiel, 2011; Rius, Potter, Aguirre, & Stachowicz, 2014b).

Our study corroborated the findings of Johannesson et al. (2018) by identifying strong genetic differentiation between shallow and deep populations of *C. intestinalis* along the west coast of Sweden. While this genomic differentiation among populations appears surprising as some deep and shallow sites are geographically close to one another, this can be explained due to the effect of the aforementioned pycnocline promoting depth‐defined divergence that has also been observed in corals (Prada & Hellberg, 2013). Admixture between deep Sweden and England, Jersey, France genotypes, as indicated by our ABC analysis, suggests that the pycnocline may not have always been the impenetrable barrier to gene flow as currently observed (Johannesson et al., 2018). Taken together, our results suggest that although historically the pycnocline may have allowed the mixing of divergent genotypes, it currently provides a strong barrier to gene flow, maintaining contemporary genomic differentiation between deep and shallow sites.

The northwest Atlantic range of *C. intestinalis* is restricted to the east coast of North America. It has been documented in eastern Canada since at least the mid‐1800s (Carver, Mallet, & Vercaemer, 2006), but its population size and range have only recently expanded (Ramsay, Davidson, Landry, & Arsenault, 2008). Our ABC analyses suggest that the origin of the Canadian sites was due to secondary contact between populations from England, Jersey, France and shallow Sweden. This is supported by ADMIXTURE analysis, which indicates high similarity between the individuals from Canada and the ones from both the English Chanel and shallow sites within the North Sea (Figure 2). In addition, the DAPC analyses indicated that Canadian individuals were similar to individuals found in shallow Swedish waters (Figure 3). This interpretation accords with previous studies showing that multiple introductions facilitate marine biological invasions (Rius et al., 2015; Simon‐Bouhet, Garcia‐Meunier, & Viard, 2006) and that recurrent introductions of large numbers of individuals explain patterns of genetic diversity within introduced ranges (Uller & Leimu, 2011). Indeed, our results do not show a noticeable change in genetic diversity between Canadian and European sites (Table 1) and are compatible with contemporary introgression among divergent English Channel and North Sea genotypes followed by multiple introductions to Canadian sites.

Heterozygote deficiency at nine of our sites (*F*_IS_ values significantly greater than zero; Table 1) could reflect either selection against heterozygotes or non‐random mating. We can reject selection against heterozygotes as we excluded loci putatively under selection, and we can also exclude selfing as self‐fertilization success is generally low in *C. intestinalis* (Bouchemousse, Lévêque, Dubois, & Viard, 2016b; Byrd & Lambert, 2000). A more likely explanation is a Wahlund effect (Wahlund, 1928), a reduction of expected heterozygosity due to mixing of two genetically differentiated populations, which has been reported in other studies (Dupont, Viard, Dowell, Wood, & Bishop, 2009; Marescaux et al., 2015) including studies of *Ciona* spp. (Hudson et al., 2016; Zhan et al., 2010).

The history of the introduction of *C. intestinalis* to the western Atlantic coast is complex, starting with historical divergence in the native range involving two groups (England, Jersey, France and deep Sweden lineages, Figure 5), which was likely due to genetic drift during a period of isolation (allopatry) in different glacial refugia. More recent historic gene flow between these populations appears to have led to the formation of the admixed genotypes found in shallow Sweden and Denmark sites. Finally, the Canada specimens originated from secondary contacts between individuals from these sites and individuals from the western North Sea and English Channel. Our findings suggest that admixture between genetically diverse native genotypes preceded successful trans‐oceanic colonization, in line with previous studies showing that genetic admixture facilitates the colonization of new habitats (Abbott, Barton, & Good, 2016). We suggest that artificial transport of species facilitates secondary contacts and intraspecific admixture among divergent native genotypes, strongly altering NIS evolutionary trajectories and influencing their ecological impacts within the introduced range.

## CONFLICT OF INTEREST

None declared,

## Data Availability

GBS data collected for this study are available as VCF files from the Dryad Digital Repository at https://doi:10.5061/dryad.wh70rxwhw.

## References

[eva12893-bib-0001] Abbott, R. J. (1992). Plant invasions, interspecific hybridization and the evolution of new plant taxa. Trends in Ecology & Evolution, 7, 401–405. 10.1016/0169-5347(92)90020-C 21236080

[eva12893-bib-0002] Abbott, R. J. , Barton, N. H. , & Good, J. M. (2016). Genomics of hybridization and its evolutionary consequences. Molecular Ecology, 25, 2325–2332. 10.1111/mec.13685 27145128

[eva12893-bib-0003] Aldred, N. , & Clare, A. S. (2014). Mini‐review: Impact and dynamics of surface fouling by solitary and compound ascidians. Biofouling, 30, 259–270. 10.1080/08927014.2013.866653 24447209

[eva12893-bib-0004] Alexander, D. H. , Novembre, J. , & Lange, K. (2009). Fast model‐based estimation of ancestry in unrelated individuals. Genome Research, 19, 1655–1664. 10.1101/gr.094052.109 19648217 PMC2752134

[eva12893-bib-0005] Bellas, J. , Beiras, R. , & Vázquez, E. (2003). A standardisation of *Ciona intestinalis* (Chordata, Ascidiacea) embryo‐larval bioassay for ecotoxicological studies. Water Research, 37, 4613–4622. 10.1016/S0043-1354(03)00396-8 14568047

[eva12893-bib-0006] Bierne, N. , Roze, D. , & Welch, J. J. (2013). Pervasive selection or is it…? Why are FST outliers sometimes so frequent? Molecular Ecology, 22, 2061–2064. 10.1111/mec.12241 23671920

[eva12893-bib-0007] Bock, D. G. , Caseys, C. , Cousens, R. D. , Hahn, M. A. , Heredia, S. M. , Hübner, S. et al. (2015). What we still don't know about invasion genetics. Molecular Ecology, 24, 2277–2297. 10.1111/mec.13032 25474505

[eva12893-bib-0008] Boubou, A. , Migeon, A. , Roderick, G. K. , Auger, P. , Cornuet, J. M. , Magalhães, S. , & Navajas, M. (2012). Test of colonisation scenarios reveals complex invasion history of the red tomato spider mite *Tetranychus evansi* . PLoS ONE, 7, e35601 10.1371/journal.pone.0035601 22539983 PMC3335100

[eva12893-bib-0009] Bouchemousse, S. , Bishop, J. D. , & Viard, F. (2016a). Contrasting global genetic patterns in two biologically similar, widespread and invasive *Ciona* species (Tunicata, Ascidiacea). Scientific Reports, 6, 24875 10.1038/srep24875 27137892 PMC4853746

[eva12893-bib-0010] Bouchemousse, S. , Lévêque, L. , Dubois, G. , & Viard, F. (2016b). Co‐occurrence and reproductive synchrony do not ensure hybridization between an alien tunicate and its interfertile native congener. Evolutionary Ecology, 30, 69–87. 10.1007/s10682-015-9788-1

[eva12893-bib-0011] Bouchemousse, S. , Liautard‐Haag, C. , Bierne, N. , & Viard, F. (2016c). Distinguishing contemporary hybridization from past introgression with post‐genomic ancestry‐informative SNPs in strongly differentiated *Ciona* species. Molecular Ecology, 25, 5527–5542. 10.1111/mec.13854 27662427

[eva12893-bib-0012] Bourne, S. D. , Hudson, J. , Holman, L. E. , & Rius, M. (2018). Marine invasion genomics: Revealing ecological and evolutionary consequences of biological invasions In Oleksiak M. F. & Rajora O. P. (Eds.), Population Genomics (pp. 1–36). Cham, Switzerland: Springer.

[eva12893-bib-0013] Byrd, J. , & Lambert, C. C. (2000). Mechanism of the block to hybridization and selfing between the sympatric ascidians *Ciona intestinalis* and *Ciona savignyi* . Molecular Reproduction and Development, 55, 109–116. 10.1002/(SICI)1098-2795(200001)55:1<109:AID-MRD15>3.0.CO;2-B 10602281

[eva12893-bib-0014] Carlton, J. T. , Chapman, J. W. , Geller, J. B. , Miller, J. A. , Carlton, D. A. , McCuller, M. I. et al. (2017). Tsunami‐driven rafting: Transoceanic species dispersal and implications for marine biogeography. Science, 357, 1402–1406. 10.1126/science.aao1498 28963256

[eva12893-bib-0015] Carver, C. , Mallet, A. , & Vercaemer, B. (2006). Biological synopsis of the solitary tunicate *Ciona intestinalis*, vol. 55, (p. 2746). Canadian Manuscript Report of Fisheries and Aquatic Sciences.

[eva12893-bib-0016] Clarke Murray, C. , Gartner, H. , Gregr, E. J. , Chan, K. , Pakhomov, E. , & Therriault, T. W. (2014). Spatial distribution of marine invasive species: Environmental, demographic and vector drivers. Diversity and Distributions, 20, 824–836. 10.1111/ddi.12215

[eva12893-bib-0017] Cornuet, J. M. , Pudlo, P. , Veyssier, J. , Dehne‐Garcia, A. , Gautier, M. , Leblois, R. et al. (2014). DIYABC v2.0: A software to make approximate Bayesian computation inferences about population history using single nucleotide polymorphism, DNA sequence and microsatellite data. Bioinformatics, 30, 1187–1189. 10.1093/bioinformatics/btt763 24389659

[eva12893-bib-0018] Cornuet, J. M. , Santos, F. , Beaumont, M. A. , Robert, C. P. , Marin, J. M. , Balding, D. J. et al. (2008). Inferring population history with DIY ABC: A user‐friendly approach to approximate Bayesian computation. Bioinformatics, 24, 2713–2719. 10.1093/bioinformatics/btn514 18842597 PMC2639274

[eva12893-bib-0019] Cristescu, M. E. (2015). Genetic reconstructions of invasion history. Molecular Ecology, 24, 2212–2225. 10.1111/mec.13117 25703061

[eva12893-bib-0020] Danecek, P. , Auton, A. , Abecasis, G. , Albers, C. A. , Banks, E. , DePristo, M. A. et al. (2011). The variant call format and VCFtools. Bioinformatics, 27, 2156–2158. 10.1093/bioinformatics/btr330 21653522 PMC3137218

[eva12893-bib-0021] Dumont, C. P. , Gaymer, C. F. , & Thiel, M. (2011). Predation contributes to invasion resistance of benthic communities against the non‐indigenous tunicate *Ciona intestinalis* . Biological Invasions, 13, 2023–2034. 10.1007/s10530-011-0018-7

[eva12893-bib-0022] Dupont, L. , Jollivet, D. , & Viard, F. (2003). High genetic diversity and ephemeral drift effects in a successful introduced mollusc (*Crepidula fornicata*: Gastropoda). Marine Ecology Progress Series, 253, 183–195. 10.3354/meps253183

[eva12893-bib-0023] Dupont, L. , Viard, F. , Dowell, M. J. , Wood, C. , & Bishop, J. D. (2009). Fine‐ and regional‐scale genetic structure of the exotic ascidian *Styela clava* (Tunicata) in southwest England, 50 years after its introduction. Molecular Ecology, 18, 442–453. 10.1111/j.1365-294X.2008.04045.x 19161467

[eva12893-bib-0024] Dybern, B. I. (1965). The life cycle of *Ciona intestinalis* (L.) f. typica in relation to the environmental temperature. Oikos, 16, 109–131. 10.2307/3564870

[eva12893-bib-0025] Dybern, B. I. (1967). The distribution and salinity tolerance of *Ciona intestinalis* (L.) f. typica with special reference to the waters around Southern Scandinavia. Ophelia, 4, 207–226. 10.1080/00785326.1967.10409621

[eva12893-bib-0026] Eaton, D. A. R. (2014). PyRAD: Assembly of de novo RADseq loci for phylogenetic analyses. Bioinformatics, 30, 1844–1849. 10.1093/bioinformatics/btu121 24603985

[eva12893-bib-0027] Elshire, R. J. , Glaubitz, J. C. , Sun, Q. , Poland, J. A. , Kawamoto, K. , Buckler, E. S. , & Mitchell, S. E. (2011). A robust, simple genotyping‐by‐sequencing (GBS) approach for high diversity species. PLoS ONE, 6, e19379 10.1371/journal.pone.0019379 21573248 PMC3087801

[eva12893-bib-0028] Estoup, A. , & Guillemaud, T. (2010). Reconstructing routes of invasion using genetic data: Why, how and so what? Molecular Ecology, 19, 4113–4130. 10.1111/j.1365-294X.2010.04773.x 20723048

[eva12893-bib-0029] Excoffier, L. , & Lischer, H. E. L. (2010). Arlequin suite ver 3.5: A new series of programs to perform population genetics analyses under Linux and Windows. Molecular Ecology Resources, 10, 564–567. 10.1111/j.1755-0998.2010.02847.x 21565059

[eva12893-bib-0030] Federman, S. , Donoghue, M. J. , Daly, D. C. , & Eaton, D. A. R. (2018). Reconciling species diversity in a tropical plant clade (*Canarium*, Burseraceae). PLoS ONE, 13, e0198882 10.1371/journal.pone.0198882 29906281 PMC6003679

[eva12893-bib-0031] Fitridge, I. , Dempster, T. , Guenther, J. , & de Nys, R. (2012). The impact and control of biofouling in marine aquaculture: A review. Biofouling, 28, 649–669. 10.1080/08927014.2012.700478 22775076

[eva12893-bib-0032] Foll, M. , & Gaggiotti, O. (2008). A genome‐scan method to identify selected loci appropriate for both dominant and codominant markers: A Bayesian perspective. Genetics, 180, 977–993. 10.1534/genetics.108.092221 18780740 PMC2567396

[eva12893-bib-0033] Gallager, S. M. , Davis, C. S. , Epstein, A. W. , Solow, A. , & Beardsley, R. C. (1996). High‐resolution observations of plankton spatial distributions correlated with hydrography in the Great South Channel, Georges Bank. Deep Sea Research Part II: Topical Studies in Oceanography, 43, 1627–1663. 10.1016/S0967-0645(96)00058-6

[eva12893-bib-0034] Gilk, S. E. , Wang, I. A. , Hoover, C. L. , Smoker, W. W. , Taylor, S. G. , Gray, A. K. , & Gharrett, A. J. (2004). Outbreeding depression in hybrids between spatially separated pink salmon, *Oncorhynchus gorbuscha*, populations: Marine survival, homing ability, and variability in family size. Environmental Biology of Fishes, 69, 287–297. 10.1023/B:EBFI.0000022888.28218.c1

[eva12893-bib-0035] Gillis, N. K. , Walters, L. J. , Fernandes, F. C. , & Hoffman, E. A. (2009). Higher genetic diversity in introduced than in native populations of the mussel *Mytella charruana*: Evidence of population admixture at introduction sites. Diversity and Distributions, 15, 784–795. 10.1111/j.1472-4642.2009.00591.x

[eva12893-bib-0036] Gulliksen, B. , & Skjæveland, S. H. (1973). The sea‐star, *Asterias rubens* L., as predator on the ascidian, *Ciona intestinalis* (L.), in Borgenfjorden, North‐Tröndelag, Norway. Sarsia, 52, 15–20. 10.1080/00364827.1973.10411228

[eva12893-bib-0037] Guzinski, J. , Ballenghien, M. , Daguin‐Thiébaut, C. , Lévêque, L. , & Viard, F. (2018). Population genomics of the introduced and cultivated Pacific kelp *Undaria pinnatifida*: Marinas‐not farms‐drive regional connectivity and establishment in natural rocky reefs. Evolutionary Applications, 11, 1582–1597. 10.1111/eva.12647 30344629 PMC6183462

[eva12893-bib-0038] Haye, P. A. , & Muñoz‐Herrera, N. C. (2013). Isolation with differentiation followed by expansion with admixture in the tunicate *Pyura chilensis* . BMC Evolutionary Biology, 13, 252 10.1186/1471-2148-13-252 24238017 PMC3840596

[eva12893-bib-0039] Hudson, J. , Viard, F. , Roby, C. , & Rius, M. (2016). Anthropogenic transport of species across native ranges: Unpredictable genetic and evolutionary consequences. Biology Letters, 12, 20160620 10.1098/rsbl.2016.0620 27729485 PMC5095196

[eva12893-bib-0040] Johannesson, K. , Ring, A.‐K. , Johannesson, K. B. , Renborg, E. , Jonsson, P. R. , & Havenhand, J. N. (2018). Oceanographic barriers to gene flow promote genetic subdivision of the tunicate *Ciona intestinalis* in a North Sea archipelago. Marine Biology, 165, 126 10.1007/s00227-018-3388-x 30100627 PMC6061499

[eva12893-bib-0041] Johansen‐Morris, A. , & Latta, R. G. (2006). Fitness consequences of hybridization between ecotypes of *Avena barbata*: Hybrid breakdown, hybrid vigor, and transgressive segregation. Evolution, 60, 1585–1595. 10.1111/j.0014-3820.2006.tb00503.x 17017059

[eva12893-bib-0042] Johnston, E. L. , Dafforn, K. A. , Clark, G. F. , Rius, M. , & Floerl, O. (2017). How anthropogenic activities affect the establishment and spread of non‐indigenous species post‐arrival. Oceanography and Marine Biology: An Annual Review, 55, 2–33.

[eva12893-bib-0043] Jombart, T. (2008). adegenet: A R package for the multivariate analysis of genetic markers. Bioinformatics, 24, 1403–1405. 10.1093/bioinformatics/btn129 18397895

[eva12893-bib-0044] Jombart, T. , Devillard, S. , & Balloux, F. (2010). Discriminant analysis of principal components: A new method for the analysis of genetically structured populations. BMC Genetics, 11, 94 10.1186/1471-2156-11-94 20950446 PMC2973851

[eva12893-bib-0045] Kaluza, P. , Kölzsch, A. , Gastner, M. T. , & Blasius, B. (2010). The complex network of global cargo ship movements. Journal of the Royal Society Interface, 7, 1093–1103. 10.1098/rsif.2009.0495 PMC2880080 20086053

[eva12893-bib-0046] Keller, L. F. , & Waller, D. M. (2002). Inbreeding effects in wild populations. Trends in Ecology & Evolution, 17, 230–241. 10.1016/S0169-5347(02)02489-8

[eva12893-bib-0047] Lambert, C. C. , & Lambert, G. (1998). Non‐indigenous ascidians in southern California harbors and marinas. Marine Biology, 130, 675–688. 10.1007/s002270050289

[eva12893-bib-0048] Lockwood, J. L. , Cassey, P. , & Blackburn, T. (2005). The role of propagule pressure in explaining species invasions. Trends in Ecology & Evolution, 20, 223–228. 10.1016/j.tree.2005.02.004 16701373

[eva12893-bib-0049] Lutz‐Collins, V. , Ramsay, A. , Quijón, P. A. , & Davidson, J. (2009). Invasive tunicates fouling mussel lines: Evidence of their impact on native tunicates and other epifaunal invertebrates. Aquatic Invasions, 4, 213–220. 10.3391/ai.2009.4.1.22

[eva12893-bib-0050] Luu, K. , Bazin, E. , & Blum, M. G. (2017). pcadapt: An R package to perform genome scans for selection based on principal component analysis. Molecular Ecology Resources, 17, 67–77. 10.1111/1755-0998.12592 27601374

[eva12893-bib-0051] Marescaux, J. , von Oheimb, K. C. M. , Etoundi, E. , von Oheimb, P. V. , Albrecht, C. , Wilke, T. , & Van Doninck, K. (2015). Unravelling the invasion pathways of the quagga mussel (*Dreissena rostriformis*) into Western Europe. Biological Invasions, 18, 245–264. 10.1007/s10530-015-1005-1

[eva12893-bib-0052] Millar, R. (1971). The biology of ascidians In Russell F. S. & Yonge M. (Eds.), Advances in marine biology, vol. 9, (pp. 1–100). London, UK: Academic Press.

[eva12893-bib-0053] Mooney, H. A. , & Cleland, E. E. (2001). The evolutionary impact of invasive species. Proceedings of the National Academy of Sciences, 98(10), 5446–5451. 10.1073/pnas.091093398 PMC33232 11344292

[eva12893-bib-0054] Nydam, M. L. , & Harrison, R. G. (2007). Genealogical relationships within and among shallow‐water *Ciona* species (Ascidiacea). Marine Biology, 151, 1839–1847. 10.1007/s00227-007-0617-0

[eva12893-bib-0055] Nydam, M. L. , Yanckello, L. M. , Bialik, S. B. , Giesbrecht, K. B. , Nation, G. K. , & Peak, J. L. (2017). Introgression in two species of broadcast spawning marine invertebrate. Biological Journal of the Linnean Society, 120, 879–890. 10.1093/biolinnean/blw012

[eva12893-bib-0056] O’Connor, T. D. , Fu, W. , Mychaleckyj, J. C. , Logsdon, B. , Auer, P. , Carlson, C. S. , … Akey, J. M. (2015). Rare variation facilitates inferences of fine‐scale population structure in humans. Molecular Biology and Evolution, 32, 653–660. 10.1093/molbev/msu326 25415970 PMC4327153

[eva12893-bib-0057] O'Leary, S. J. , Puritz, J. B. , Willis, S. C. , Hollenbeck, C. M. , & Portnoy, D. S. (2018). These aren't the loci you're looking for: Principles of effective SNP filtering for molecular ecologists. Molecular Ecology, 27, 3193–3206. 10.1111/mec.14792 29987880

[eva12893-bib-0058] Pérez, J. E. , Nirchio, M. , Alfonsi, C. , & Muñoz, C. (2006). The biology of invasions: The genetic adaptation paradox. Biological Invasions, 8, 1115–1121. 10.1007/s10530-005-8281-0

[eva12893-bib-0059] Petersen, J. K. , & Svane, I. (1995). Larval dispersal in the ascidian *Ciona intestinalis* (L.). Evidence for a closed population. Journal of Experimental Marine Biology and Ecology, 186, 89–102. 10.1016/0022-0981(94)00157-9

[eva12893-bib-0060] Pineda, M. C. , López‐Legentil, S. , & Turon, X. (2011). The whereabouts of an ancient wanderer: Global phylogeography of the solitary ascidian *Styela plicata* . PLoS ONE, 6, e25495 10.1371/journal.pone.0025495 21966535 PMC3179514

[eva12893-bib-0061] Prada, C. , & Hellberg, M. E. (2013). Long prereproductive selection and divergence by depth in a Caribbean candelabrum coral. Proceedings of the National Academy of Science USA, 110, 3961–3966. 10.1073/pnas.1208931110 PMC3593850 23359716

[eva12893-bib-0062] Pritchard, J. K. , Stephens, M. , & Donnelly, P. (2000). Inference of population structure using multilocus genotype data. Genetics, 155, 945–959. 10835412 10.1093/genetics/155.2.945 PMC1461096

[eva12893-bib-0086] R Development Core Team (2017). R: A language and environment for statistical computing. Vienna, Austria: R Foundation for Statistical Computing.

[eva12893-bib-0063] Ramsay, A. , Davidson, J. , Landry, T. , & Arsenault, G. (2008). Process of invasiveness among exotic tunicates in Prince Edward Island, Canada. Biological Invasions, 10, 1311–1316. 10.1007/s10530-007-9205-y

[eva12893-bib-0064] Rees, H. L. , Waldock, R. , Matthiessen, P. , & Pendle, M. A. (2001). Improvements in the epifauna of the Crouch Estuary (United Kingdom) following a decline in TBT concentrations. Marine Pollution Bulletin, 42, 137–144. 10.1016/S0025-326X(00)00119-3 11381884

[eva12893-bib-0065] Renborg, E. , Johannesson, K. , & Havenhand, J. (2014). Variable salinity tolerance in ascidian larvae is primarily a plastic response to the parental environment. Evolutionary Ecology, 28, 561–572. 10.1007/s10682-013-9687-2

[eva12893-bib-0066] Reusch, T. B. , Bolte, S. , Sparwel, M. , Moss, A. G. , & Javidpour, J. (2010). Microsatellites reveal origin and genetic diversity of Eurasian invasions by one of the world's most notorious marine invader, *Mnemiopsis leidyi* (Ctenophora). Molecular Ecology, 19, 2690–2699. 10.1111/j.1365-294X.2010.04701.x 20561193

[eva12893-bib-0067] Rius, M. , Clusella‐Trullas, S. , McQuaid, C. D. , Navarro, R. A. , Griffiths, C. L. , Matthee, C. A. et al. (2014a). Range expansions across ecoregions: Interactions of climate change, physiology and genetic diversity. Global Ecology and Biogeography, 23, 76–88. 10.1111/geb.12105

[eva12893-bib-0068] Rius, M. , & Darling, J. A. (2014). How important is intraspecific genetic admixture to the success of colonising populations? Trends in Ecology & Evolution, 29, 233–242. 10.1016/j.tree.2014.02.003 24636862

[eva12893-bib-0069] Rius, M. , Heasman, K. G. , & McQuaid, C. D. (2011). Long‐term coexistence of non‐indigenous species in aquaculture facilities. Marine Pollution Bulletin, 62, 2395–2403. 10.1016/j.marpolbul.2011.08.030 21945559

[eva12893-bib-0070] Rius, M. , Potter, E. E. , Aguirre, J. D. , & Stachowicz, J. J. (2014b). Mechanisms of biotic resistance across complex life cycles. Journal of Animal Ecology, 83, 296–305. 10.1111/1365-2656.12129 24350679

[eva12893-bib-0071] Rius, M. , Turon, X. , Bernardi, G. , Volckaert, F. A. M. , & Viard, F. (2015). Marine invasion genetics: From spatio‐temporal patterns to evolutionary outcomes. Biological Invasions, 17, 869–885. 10.1007/s10530-014-0792-0

[eva12893-bib-0072] Rius, M. , Turon, X. , Ordóñez, V. , & Pascual, M. (2012). Tracking invasion histories in the sea: Facing complex scenarios using multilocus data. PLoS ONE, 7, e35815 10.1371/journal.pone.0035815 22545140 PMC3335797

[eva12893-bib-0073] Roman, J. , & Darling, J. A. (2007). Paradox lost: Genetic diversity and the success of aquatic invasions. Trends in Ecology & Evolution, 22, 454–464. 10.1016/j.tree.2007.07.002 17673331

[eva12893-bib-0074] Simon‐Bouhet, B. , Garcia‐Meunier, P. , & Viard, F. (2006). Multiple introductions promote range expansion of the mollusc *Cyclope neritea* (Nassariidae) in France: Evidence from mitochondrial sequence data. Molecular Ecology, 15, 1699–1711. 10.1111/j.1365-294X.2006.02881.x 16629822

[eva12893-bib-0075] Svane, I. (1983). Ascidian reproductive patterns related to long‐term population dynamics. Sarsia, 68, 249–255. 10.1080/00364827.1983.10420578

[eva12893-bib-0076] Svane, I. , & Havenhand, J. (1993). Spawning and dispersal in *Ciona intestinalis* (L.). Marine Ecology, 14, 53–66. 10.1111/j.1439-0485.1993.tb00364.x

[eva12893-bib-0077] Uller, T. , & Leimu, R. (2011). Founder events predict changes in genetic diversity during human‐mediated range expansions. Global Change Biology, 17, 3478–3485. 10.1111/j.1365-2486.2011.02509.x

[eva12893-bib-0078] Verhoeven, K. J. F. , Macel, M. , Wolfe, L. M. , & Biere, A. (2011). Population admixture, biological invasions and the balance between local adaptation and inbreeding depression. Proceedings of the Royal Society B: Biological Sciences, 278, 2–8. 10.1098/rspb.2010.1272 PMC2992731 20685700

[eva12893-bib-0079] Wagner, N. K. , Ochocki, B. M. , Crawford, K. M. , Compagnoni, A. , & Miller, T. E. (2017). Genetic mixture of multiple source populations accelerates invasive range expansion. Journal of Animal Ecology, 86, 21–34. 10.1111/1365-2656.12567 27363388

[eva12893-bib-0080] Wahlund, S. (1928). Zusammensetzung von populationen und korrelationserscheinungen vom standpunkt der vererbungslehre aus betrachtet. Hereditas, 11, 65–106. 10.1111/j.1601-5223.1928.tb02483.x

[eva12893-bib-0081] White, O. W. , Reyes‐Betancort, A. , Chapman, M. A. , & Carine, M. A. (2018). Independent homoploid hybrid speciation events in the Macaronesian endemic genus *Argyranthemum* . Molecular Ecology, 27, 4856–4874. 10.1111/mec.14889 30281862

[eva12893-bib-0082] Whitlock, M. C. , & Lotterhos, K. E. (2015). Reliable detection of loci responsible for local adaptation: Inference of a null model through trimming the distribution of F(ST). The American Naturalist, 186(S1), S24–S36. 10.1086/682949 26656214

[eva12893-bib-0083] Yoshida, T. , Goka, K. , Ishihama, F. , Ishihara, M. , & Kudo, S.‐I. (2007). Biological invasion as a natural experiment of the evolutionary processes: Introduction of the special feature. Ecological Research, 22, 849–854. 10.1007/s11284-007-0435-3

[eva12893-bib-0084] Zhan, A. , Briski, E. , Bock, D. G. , Ghabooli, S. , & MacIsaac, H. J. (2015). Ascidians as models for studying invasion success. Marine Biology, 162, 2449–2470. 10.1007/s00227-015-2734-5

[eva12893-bib-0085] Zhan, A. , Macisaac, H. J. , & Cristescu, M. E. (2010). Invasion genetics of the *Ciona intestinalis* species complex: From regional endemism to global homogeneity. Molecular Ecology, 19, 4678–4694. 10.1111/j.1365-294X.2010.04837.x 20875067

