# Supplementary material for: Secondary contacts and genetic admixture shape colonization by an amphiatlantic epibenthic invertebrate
Source: Evol Appl. 2019 Dec 3;13(3):600–12. doi: 10.1111/eva.12893 (PMC7045719; doi:10.1111/eva.12893)
Supplement: Supplementary file 1 [file EVA-13-600-s001.docx]

**Supplementary material**

**Table S1.** Pairwise population comparisons of F_ST_ values and associated P values.

|  | VAT | GUL | JAM_D | KAV | BUH | KOS | BRA | LIN | POR | JAM_S | FIS | DEN | HPL | TNQ | JER | STM | CAN_1 | CAN_2 |
| --- | --- | --- | --- | --- | --- | --- | --- | --- | --- | --- | --- | --- | --- | --- | --- | --- | --- | --- |
| VAT |  | 0.000 | 0.000 | 0.000 | 0.000 | 0.000 | 0.000 | 0.000 | 0.000 | 0.000 | 0.000 | 0.000 | 0.000 | 0.000 | 0.000 | 0.000 | 0.000 | 0.000 |
| GUL | *0.166* |  | 0.000 | 0.000 | 0.000 | 0.000 | 0.000 | 0.000 | 0.000 | 0.000 | 0.000 | 0.000 | 0.000 | 0.000 | 0.000 | 0.000 | 0.000 | 0.000 |
| JAM_D | *0.136* | *0.103* |  | 0.841 | 0.000 | 0.000 | 0.000 | 0.000 | 0.000 | 0.000 | 0.000 | 0.000 | 0.000 | 0.000 | 0.000 | 0.000 | 0.000 | 0.000 |
| KAV | *0.130* | *0.089* | -0.002 |  | 0.000 | 0.000 | 0.000 | 0.000 | 0.000 | 0.000 | 0.000 | 0.000 | 0.000 | 0.000 | 0.000 | 0.000 | 0.000 | 0.000 |
| BUH | *0.150* | *0.135* | *0.106* | *0.092* |  | 0.007 | 0.005 | 0.005 | 0.000 | 0.000 | 0.000 | 0.000 | 0.000 | 0.000 | 0.000 | 0.000 | 0.000 | 0.000 |
| KOS | *0.183* | *0.167* | *0.135* | *0.126* | 0.028 |  | 0.904 | 0.642 | 0.000 | 0.000 | 0.000 | 0.000 | 0.000 | 0.000 | 0.000 | 0.000 | 0.000 | 0.000 |
| BRA | *0.187* | *0.167* | *0.123* | *0.120* | 0.030 | -0.004 |  | 0.006 | 0.000 | 0.000 | 0.000 | 0.000 | 0.000 | 0.000 | 0.000 | 0.000 | 0.000 | 0.000 |
| LIN | *0.194* | *0.200* | *0.166* | *0.150* | 0.031 | 0.000 | 0.019 |  | 0.000 | 0.000 | 0.000 | 0.000 | 0.000 | 0.000 | 0.000 | 0.000 | 0.000 | 0.000 |
| POR | *0.255* | *0.259* | *0.227* | *0.205* | *0.100* | *0.055* | *0.073* | *0.072* |  | 0.000 | 0.000 | 0.000 | 0.000 | 0.000 | 0.000 | 0.000 | 0.000 | 0.000 |
| JAM_S | *0.166* | *0.159* | *0.123* | *0.115* | *0.061* | *0.078* | *0.073* | *0.094* | *0.092* |  | 0.000 | 0.000 | 0.000 | 0.000 | 0.000 | 0.000 | 0.000 | 0.000 |
| FIS | *0.214* | *0.198* | *0.177* | *0.160* | *0.082* | *0.077* | *0.070* | *0.085* | *0.063* | *0.063* |  | 0.000 | 0.000 | 0.000 | 0.000 | 0.000 | 0.000 | 0.000 |
| DEN | *0.207* | *0.204* | *0.173* | *0.166* | *0.138* | *0.137* | *0.120* | *0.152* | *0.160* | *0.124* | *0.136* |  | 0.000 | 0.000 | 0.000 | 0.000 | 0.000 | 0.000 |
| HPL | *0.175* | *0.198* | *0.168* | *0.171* | *0.160* | *0.165* | *0.158* | *0.175* | *0.209* | *0.158* | *0.194* | *0.154* |  | 0.000 | 0.000 | 0.000 | 0.000 | 0.000 |
| TNQ | *0.149* | *0.183* | *0.147* | *0.144* | *0.129* | *0.147* | *0.142* | *0.159* | *0.172* | *0.116* | *0.158* | *0.130* | *0.060* |  | 0.654 | 0.031 | 0.000 | 0.000 |
| JER | *0.158* | *0.197* | *0.165* | *0.160* | *0.149* | *0.156* | *0.150* | *0.170* | *0.189* | *0.138* | *0.178* | *0.140* | *0.059* | 0.001 |  | 0.001 | 0.000 | 0.000 |
| STM | *0.145* | *0.192* | *0.137* | *0.141* | *0.136* | *0.153* | *0.144* | *0.163* | *0.185* | *0.133* | *0.170* | *0.139* | *0.049* | 0.016 | 0.025 |  | 0.000 | 0.000 |
| CAN_1 | *0.147* | *0.148* | *0.108* | *0.105* | *0.063* | *0.066* | *0.064* | *0.074* | *0.127* | *0.090* | *0.110* | *0.110* | *0.095* | *0.073* | *0.081* | *0.067* |  | 0.000 |
| CAN_2 | *0.162* | *0.192* | *0.138* | *0.140* | *0.082* | *0.055* | *0.064* | *0.067* | *0.126* | *0.107* | *0.124* | *0.119* | *0.116* | *0.088* | *0.091* | *0.090* | *0.033* |  |

*Note*: F_ST_ values are below the diagonal and P values above the diagonal. Values in *italics* are significant after Bonferroni correction. Site abbreviations are as in Table 1. Note that the dataset used is with F_ST_ outlier loci recovered from both BayeScan and pcadapt removed (14 loci removed).

**Table S2.** Analysis of Molecular Variance under the assumption of four clusters as identified by ADMIXTURE. Note that the dataset used is with F_ST_ outlier loci recovered from both BayeScan and pcadapt removed (14 loci removed).

| Source of variation | d.f. | Sum of squares | Variance components | Percentage of variation | Fixation Indices |
| --- | --- | --- | --- | --- | --- |
| Among groups | 3 | 744.83 | 1.55 Va | 8.14 | F_CT_ = 0.081*** |
| Among sites within groups | 14 | 748.36 | 1.29 Vb | 6.78 | F_SC_ = 0.074*** |
| Within sites | 512 | 8279.13 | 16.17 Vc | 85.08 | F_ST_ = 0.149*** |

*Note*: Clusters are 1) Deep Sweden, 2) Shallow Sweden (except JAM_S) + CAN_1 + CAN_2, 3) JAM_S + DEN, 4) HPL + TNQ + JER + STM. *** *P* < 0.001.

**Table S3.** Analysis of Molecular Variance under the assumption of three clusters as identified by DAPC. Note that the dataset used is with F_ST_ outlier loci recovered from both BayeScan and pcadapt removed (14 loci removed).

| Source of variation | d.f. | Sum of squares | Variance components | Percentage of variation | Fixation Indices |
| --- | --- | --- | --- | --- | --- |
| Among groups | 2 | 628.68 | 1.66 Va | 8.61 | F_CT_ = 0.086*** |
| Among sites within groups | 15 | 864.51 | 1.41 Vb | 7.35 | F_SC_ = 0.080*** |
| Within sites | 512 | 8279.13 | 16.17 Vc | 84.04 | F_ST_ = 0.160*** |

*Note*: Clusters are 1) Deep Sweden, 2) Shallow Sweden + CAN_1 + CAN_2 + DEN, 3) HPL + TNQ + JER + STM. *** *P* < 0.001.

**Table S4.** Analysis of Molecular Variance under the assumption of two clusters (shallow artificial v shallow natural sites). Note that the dataset used is with F_ST_ outlier loci recovered from both BayeScan and pcadapt removed (14 loci removed).

| Source of variation | d.f. | Sum of squares | Variance components | Percentage of variation | Fixation Indices |
| --- | --- | --- | --- | --- | --- |
| Among groups | 1 | 31.40 | -0.16 Va | -0.98 | F_CT_ = -0.010 |
| Among sites within groups | 5 | 240.17 | 1.08 Vb | 6.61 | F_SC_ = 0.066*** |
| Within populations | 410 | 3150.70 | 15.37 Vc | 94.36 | F_ST_ = 0.056*** |

*Note*: *** *P* < 0.001.

**Table S5**. Prior distribution parameters describing the set of scenarios investigated using DIYABC**.**

| Parameter | Distribution | Min | Max |
| --- | --- | --- | --- |
| *Effective population size* | |  |  |
| N1, N2, N3, N4 | Uniform | 10000000 | 10000000 |
|  |  |  |  |
| *Time of events* | |  |  |
| t1, t2, t3 | Uniform | 10 | 10000000 |
|  |  |  |  |
| *Admixture rate* | |  |  |
| r1 | Uniform | 0.001 | 0.999 |

*Note*: The time priors were constrained (t1<t2<t3) and included split or admixed events, N1,N2,N3,N4: effective population size of the DIYABC groupings (see methods and Table 1).

**Table S6.** Posterior probabilities and 95% confidence intervals of the sets of DIYABC scenarios. Note that the dataset used is with F_ST_ outlier loci recovered from both BayeScan and pcadapt removed (14 loci removed).

|  | Scenario | Posterior probability | Confidence interval |
| --- | --- | --- | --- |
| Scenario set 1 | 1 | 0.0000 | 0.0000,0.3467 |
|  | 2 | 0.0000 | 0.0000,0.3467 |
|  | *3* | *0.9961* | *0.8007,0.8990* |
|  | 4 | 0.0039 | 0.0000,0.3496 |
|  | 5 | 0.0000 | 0.0000,0.3467 |
|  | 6 | 0.0000 | 0.0000,0.3467 |
|  | 7 | 0.0000 | 0.0000,0.3477 |
| Scenario set 2 | 1 | 0.0008 | 0.0006,0.0010 |
|  | *2* | *0.9984* | *0.9980,0.9988* |
|  | 3 | 0.0008 | 0.0000,0.2422 |
| Scenario set 3 | 1 | 0.0000 | 0.0000,0.0011 |
|  | 2 | 0.1041 | 0.0953,0.1130 |
|  | 3 | 0.0531 | 0.0491,0.0571 |
|  | 4 | 0.0014 | 0.0004,0.0024 |
|  | *5* | *0.8413* | *0.8319,0.8506* |

*Note*: Scenarios and scenario sets outlined in Fig S2. Scenarios in *italics* had the highest posterior probability without overlapping confidence intervals.

**Table S7.** Model checking in DIYABC for scenario 5. Summary statistic abbreviations include variance of non-zero values for genic diversities (HV1_1), F_ST_ distances (FV1_1), Nei’s distances (NV1_1) and admixture estimates (AV1_1). Asterisks represent proportions lower than 1% or greater than 99% (**), or lower than 0.1% or greater than 99.9% (***). Note that the dataset used is with F_ST_ outlier loci recovered from both BayeScan and pcadapt removed (14 loci removed).

| Summary statistic | Observed value | Proportion (simulated > observed) |
| --- | --- | --- |
| HV1_1_1 | 0.022 | 0 (***) |
| HV1_1_2 | 0.023 | 0 (***) |
| HV1_1_3 | 0.024 | 0.002 (**) |
| HV1_1_4 | 0.023 | 0.170 |
| FV1_1_1&2 | 0.008 | 1 (***) |
| FV1_1_1&3 | 0.015 | 0.662 |
| FV1_1_1&4 | 0.015 | 0.449 |
| FV1_1_2&3 | 0.018 | 0.703 |
| FV1_1_2&4 | 0.014 | 0.333 |
| FV1_1_3&4 | 0.016 | 0.421 |
| NV1_1_1&2 | 0.003 | 0.994 (**) |
| NV1_1_1&3 | 0.002 | 0.500 |
| NV1_1_1&4 | 0.005 | 0.428 |
| NV1_1_2&3 | 0.007 | 0.849 |
| NV1_1_2&4 | 0.006 | 0.333 |
| NV1_1_3&4 | 0.006 | 0.406 |
| AV1_1_4&1&2 | 0.107 | 1 (***) |
| AV1_1_4&1&3 | 0.086 | 0.533 |
| AV1_1_4&2&3 | 0.097 | 0.605 |

**
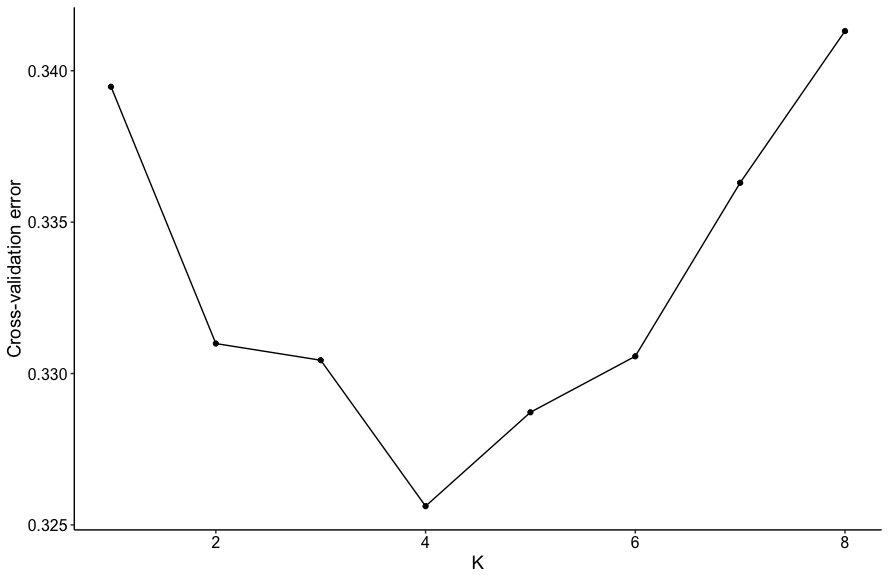
**

**Fig. S1.** Cross validation error estimate from the ADMIXTURE analysis showing K = 4 as an appropriate modelling choice. Note that the dataset used is with F_ST_ outlier loci recovered from both BayeScan and pcadapt removed (14 loci removed).

**A)**


**B)**

**C)**

**Fig S2.** Prior checking for each DIYABC run. A principal component analysis was performed in the space of summary statistics on 100,000 simulated data sets and the observed data was added on each plane. Fig. 2SA: Scenario set 1, Fig. 2SB: Scenario set 2, and Fig. S2C: Scenario set 3. Note that the dataset used is with F_ST_ outlier loci recovered from both BayeScan and pcadapt removed (14 loci removed).

**A)**

**B)**

**C)**

**Fig. S3.** DIYABC scenarios used to assess colonisation history within the northeast Atlantic (Fig. S2A, S2B) and across the north Atlantic (Fig. S2C). Scenarios in Fig. S2A (scenario set 1) assessed the ancestral populations within the northeast Atlantic and included all possible combinations (six) of an initial divergence between two groups, followed by divergence from the third group, as well as one scenario where the three groups diverged at the same time. As scenario 3 was deemed the most likely (see Results), this was used as the basis for Fig. S2B (scenario set 2), where scenarios included: 1) Initial divergence between EJF and DS, followed by divergence from EJF to form SS; 2) Initial divergence between EJF and DS, followed by secondary contact and admixture between EJF and DS which lead to SS; and 3) Initial divergence between DS and an unsampled population, which them diverged to become EJF, with SS diverging from DS. As scenario 2 was deemed the most likely (see Results), this was used as the basis for Fig. S2C (scenario set 3) which assessed the colonisation history of Canada. Scenario set 3 included CAN diverging from 1) DS; 2) SS; 3) EJF; 4) an admixture event between DS and SS; 5) and admixture event between DS and EJF. Abbreviations are shallow Sweden sites (SS), Canada sites (CAN), the Denmark site (DEN), England, Jersey, and France sites (EJF), and deep Sweden sites (DS).

**Fig. S4.** The first two axes of the PCA as part of the model checking function of DIYABC. Small yellow circles represent datasets simulated from priors, large filled yellow circles represent datasets simulated from posteriors and the large purple circle represents the observed dataset. Note that the dataset used is with F_ST_ outlier loci recovered from both BayeScan and pcadapt removed (14 loci removed).

**Appendix A: Genomic analyses excluding all outlier loci**

In order to consider all possible F_ST_ outlier loci, we ran all of the genomic analyses described in the main text but this time using using a dataset with all F_ST_ outlier loci (77 in total). These are the outlier loci identified by either BayeScan and / or pcadapt. This leads to a final dataset of 1,590 loci from the 265 individuals sequenced.

We found that values of F_IS_ ranged from 0.033 to 0.140 (Table A1). Whilst F_IS_ values are similar to the analyses included in the main text, only four sites showed no signs of deviation from Hardy-Weinberg equilibrium, compared to nine sites when only 14 outlier loci were excluded. Expected heterozygosity ranged from 0.193 to 0.279 (Table A1).

When we ran the ADMIXTURE and DAPC analyses, we found very similar patterns using both datasets (Fig. A1 and A2A). However, when we performed a DAPC with *a priori* population information on the dataset with 77 outlier loci, we found the site in Denmark (DEN) to be separated from all other samples (Fig. A2B). When this single sampling site was excluded, the pattern results then matched what is reported in Fig. 3B (Fig. A2C).

Pairwise site comparisons of F_ST_ followed the exact same pattern in both datasets, with 143 out of 153 pairwise site comparisons for F_ST_ being significant (93% of comparisons). In addition, there were also no differences in the results of the AMOVAs.

When we ran the Approximate Bayesian Method in the dataset of 77 outlier loci, the first two scenario sets recovered the same scenarios with the highest support as in the main text (i.e. the analysis including the northeast Atlantic sample sites, original divergence was between deep Sweden and England, Jersey, and France [logistic estimate of posterior probability P=0.9795, CI=0.9720, 0.9870]; the origin of the shallow Sweden population involved admixture between Deep Sweden and England, Jersey, and France [P=0.9961, CI=0.9953, 0.9969]). Regarding scenario set 3, we found again evidence for a recent admixture between EJF and SS (P=0.4487, CI=0.4285, 4689) but confidence intervals of this scenario overlapped with those of the scenario presenting Canadian individuals being introduced purely by genotypes from shallow Sweden (i.e. Scenario 2 in Fig. S3C; P=0.4775, CI=0.4587, 0.4964).

**Table A1.** F_IS_ values (values in italics are statistically significant [P<0.05]) and population mean expected heterozygosity (H_E_) for all sites using the dataset with all putative F_ST_ outlier loci excluded. Note that the dataset used is with F_ST_ outlier loci recovered from either BayeScan and pcadapt removed (77 loci removed).

| Country | Site Name | Code | F_IS_ | H_E_ |
| --- | --- | --- | --- | --- |
| Sweden | Vattenholmen | VAT | *0.078* | 0.211 |
|  | Gåseklåvan | GUL | *0.116* | 0.260 |
|  | Jämningarna | JAM_D | 0.109 | 0.279 |
|  | Kåvra | KAV | *0.127* | 0.221 |
|  | Burholmen | BUH | *0.140* | 0.193 |
|  | South Koster | KOS | *0.081* | 0.226 |
|  | Brattskär | BRA | *0.098* | 0.233 |
|  | Lindholmen | LIN | *0.083* | 0.241 |
|  | Porsholmen | POR | 0.058 | 0.231 |
|  | Jämningarna | JAM_S | 0.033 | 0.200 |
|  | Fiskebäckskil | FIS | 0.070 | 0.236 |
| Denmark | Limfjord | DEN | *0.100* | 0.215 |
| England | Hartlepool | HPL | *0.085* | 0.222 |
|  | Town Quay | TNQ | *0.123* | 0.225 |
| Jersey | St. Helier | JER | *0.092* | 0.214 |
| France | St. Malo | STM | *0.120* | 0.226 |
| Canada | Yarmouth | YAM | *0.105** | 0.220* |
|  | Shelburne | SB |  |  |
|  | Brudenell River | BR | 0.048§ | 0.255§ |
|  | Sydney | SD |  |  |

*Note*: * refers to samples merged and known as CAN_1 (Yarmouth and Shelburne), § refers to merged samples known as CAN_2 (Brudenell River and Sydney).

**Figure A1**. ADMIXTURE plots representing all sampled populations of *Ciona intestinalis*, using the dataset with all F_ST_ outlier recovered from either BayeScan and pcadapt removed (77 loci removed). The main regions are highlighted above. The different colours represent putative genetic clusters with K ranging from 2 to 7, with K=4 being found to be the most optimal value.

**
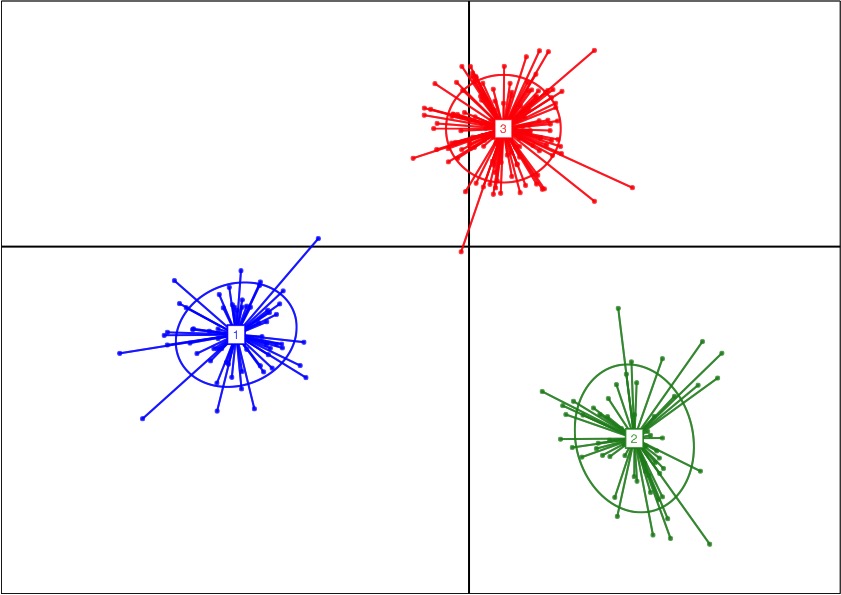
A)**

**B)**


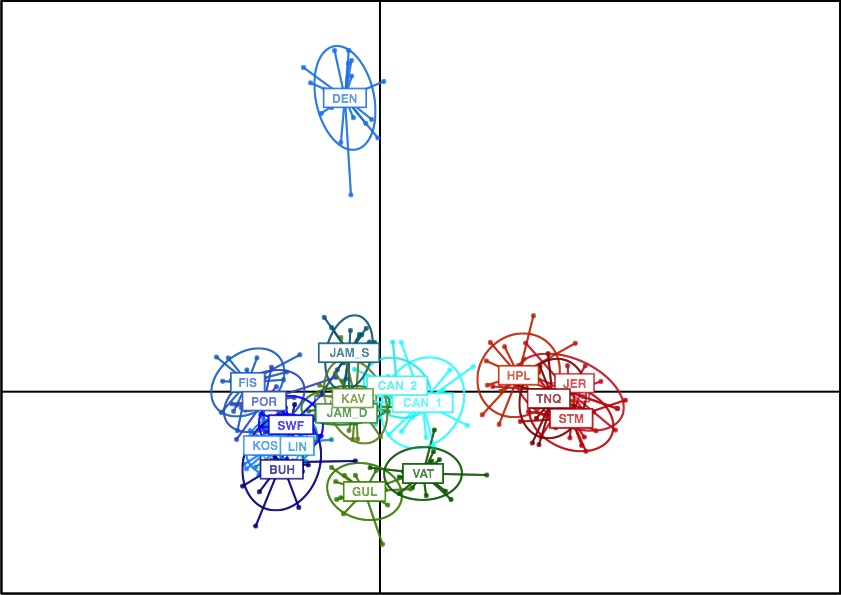


**C)**


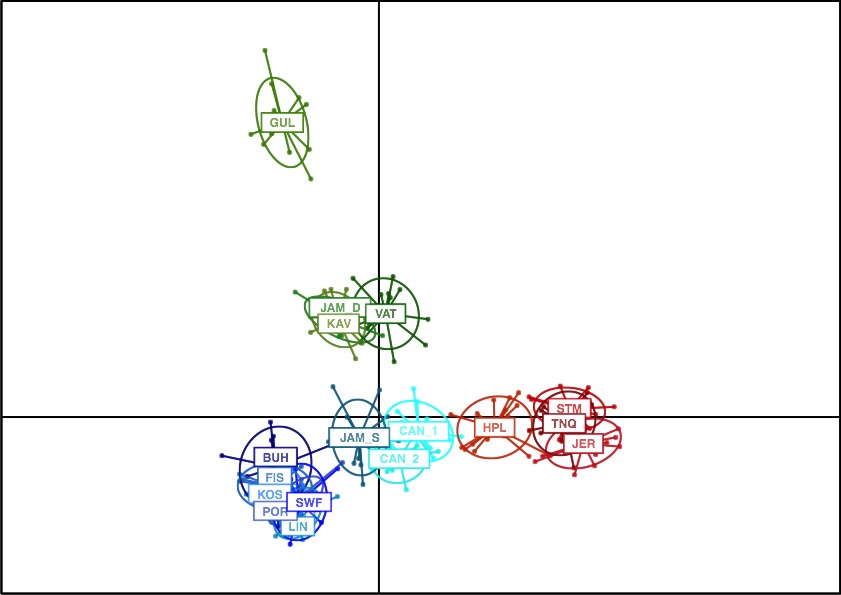


**Figure A2.** Discriminant Analysis of Principal Components with all putative F_ST_ outlier loci (77) excluded. (A) Discriminant Analysis of Principal Components using unlinked loci with no *a priori* population information. (B) Discriminant Analysis of Principal Components using unlinked loci with *a priori* population information. (C) Discriminant Analysis of Principal Components using unlinked loci with *a priori* population information excluding site DEN. Site abbreviation as in Table 1. Sites in (A) are assigned to clusters as follows; Cluster 1: : FIS, KOS, BRA, LIN, POR, BUH, JAM_S, DEN, CAN_1, CAN_2, and eight individuals from BUH; Cluster 2; JER, TNQ, HPL, STM ;Cluster 3: VAT, JAM_D, GUL, KAV, and eight individuals from BUH.
